# Pathology of inflammatory diseases of the nervous system: Human disease versus animal models

**DOI:** 10.1002/glia.23726

**Published:** 2019-10-12

**Authors:** Hans Lassmann

**Affiliations:** ^1^ Institut fur Hirnforschung Medical University of Vienna Wien Austria

**Keywords:** astrocytes, brain inflammation, lymphocytes, macrophages, microglia, multiple sclerosis

## Abstract

Numerous recent studies have been performed to elucidate the function of microglia, macrophages, and astrocytes in inflammatory diseases of the central nervous system. Regarding myeloid cells a core pattern of activation has been identified, starting with the activation of resident homeostatic microglia followed by recruitment of blood borne myeloid cells. An initial state of proinflammatory activation is at later stages followed by a shift toward an‐anti‐inflammatory and repair promoting phenotype. Although this core pattern is similar between experimental models and inflammatory conditions in the human brain, there are important differences. Even in the normal human brain a preactivated microglia phenotype is evident, and there are disease specific and lesion stage specific differences in the contribution between resident and recruited myeloid cells and their lesion state specific activation profiles. Reasons for these findings reside in species related differences and in differential exposure to different environmental cues. Most importantly, however, experimental rodent studies on brain inflammation are mainly focused on autoimmune encephalomyelitis, while there is a very broad spectrum of human inflammatory diseases of the central nervous system, triggered and propagated by a variety of different immune mechanisms.

## Graphical abstract

Inflammation in the CNS is driven by disease specific immunological mechanisms.

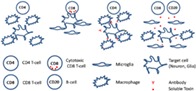

Patterns of myeloid cell activation depend upon disease specific immune mechanisms.The function of myeloid cells is determined by the lesion stage and context.

## 1 INTRODUCTION

Inflammatory diseases of the central nervous system comprise a broad spectrum of infection related or putative autoimmune disorders, characterized by the presence of inflammatory infiltrates in the brain and spinal cord and immune mediated tissue injury (Höftberger & Lassmann, 2017a, 2017b; Kuhlmann, Lassmann, & Brück, 2008; Table 1). In general, the inflammatory process involves mechanisms of adaptive immunity, reflected by the abundant presence of T‐ and B‐lymphocytes and their local activation, proliferation, and clonal expansion within the lesions. Depending upon the recognition of their specific antigen within the central nervous system, disease typical patterns of tissue destruction are seen, such as for instance selective demyelination in multiple sclerosis (Lassmann, Brück, & Lucchinetti, 2007; Lucchinetti et al., 2000), neuronal damage in Rasmussen's encephalitis (Bien et al., 2002), or astrocytic injury in neuromyelitis optica (Misu et al., 2013). Antibodies alone with some exceptions (Hillebrand et al., 2019; Höftberger & Lassmann, 2017a) are not capable to induce an inflammatory brain disease by themselves, since they do not reach the brain and spinal cord tissue in sufficient amounts through an intact blood brain barrier, but they are instrumental in augmenting and modifying inflammatory reactions in the central nervous system, induced by T‐lymphocytes (Bradl et al., 2009; Linington et al., 1988).

**Table 1:** Key features of inflammatory lesions in the CNS, mediated by different immunological mechanisms

Mechanism/references	Model	Human disease	Pathology	Microglia reaction	Astrocyte reaction
CD4^+^ T‐cells/1–5	EAE induced with MOG peptide; passive T‐cell transfer	ADEM, Clippers,	Inflammation, minor perivascular tissue damage	Moderate microglia activation; macrophage recruitment;	Transient activation
CD4^+^ T‐cells, innate immune activation/6–7	Chronic MOG peptide EAE in NOD mice	ADEM	Inflammation, perivascular lesions with demyelination and neurodegeneration	Early microglia activation, macrophage recruitment and activation	Activation; AG dystrophy in severely inflamed areas; glial scar formation
CD4^+^ T‐cells and pathogenic autoantibodies/8–12	Chronic EAE with full length MOG protein; passive transfer of T‐cells and pathogenic antibodies (anti‐MOG, anti‐AQP4)	MOG antibody associated inflammatory demyelinating disease; neuromyelitis optica spectrum disorders (NMOSD)	Chronic inflammation; confluent plaques with selective primary demyelination (MOG) or astrocytopathy followed by demyelination and neurodegeneration	Early microglia activation; macrophage recruitment; complement and macrophage associated tissue damage	Activation; antibody mediated astrocyte destruction in the presence of anti‐AQP4 antibodies; fibrillary gliosis in inactive lesions
CD8^+^ T‐cells/13–21	CD8^+^ T‐cell transfer in TG animals, expressing T‐cell antigen in specific CNS cells	Paraneoplastic encephalitis, Rasmussen's encephalitis Narcolepsy, diabetes insipidus, Multiple sclerosis?	Acute/subacute inflammation, selective destruction of specific target cells by cytotoxic T‐cells;	Proinflammatory microglia activation, little recruitment of peripheral myeloid cells	Activation in fresh lesions, glial scaring in late lesions
CD8^+^ T‐cells and CD20^+^ B‐cells/22–24	No model available	Multiple sclerosis	Chronic inflammation, primary demyelination, neurodegeneration	Proinflammatory microglia activation in initial lesions; recruitment of peripheral myeloid cells at later lesion stages	Protoplasmatic activation in early lesions (Creutzfeldt Peters cells), astrocyte dystrophy in severely inflamed areas, astrocytic scar formation in late lesion stages
Innate immunity 25–27	Bacterial meningitis/encephalitis; LPS injection into CNS; EAE in NOD mice	Bacterial meningitis; chronic inflammatory conditions	Inflammation dominated by macrophages and granulocytes; moderate to minor lymphocyte recruitment; nonselective tissue damage in CNS	Microglia activation associated with neurodegeneration	Protoplasmatic astrocyte activation in early lesions; astrocyte dystrophy in severely inflamed areas; astrocytic scar in late lesions.

*Note*: The table compares the key features of inflammatory lesions in the central nervous system mediated by different T‐cell populations, B‐cells, and innate immunity in rodent models and humans, also describing the reaction patterns of microglia/macrophages and astrocytes. References 1: Ajami et al. (2018), 2: Ben‐Nun, Wekerle, and Cohen (1981), Lassmann et al. (1993), 4: Wolf et al. (2018), 5: Blaabjerg et al. (2016). 6: Mayo et al. (2016), 7: Basso et al. (2008). 8: Bradl and Lassmann (2014), 9: Linington, Bradl, Lassmann, Brunner, and Vass (1988), 10: Misu et al. (2013), 11: Pohl et al., 2013, 12: Storch et al. (1998). 13: Bien et al. (2002), 14: Cabarrocas, Bauer, Piaggio, Liblau, and Lassmann (2003), 15: Dauvilliers et al. (2013), 16: Ji, Perchellet, and Goverman (2010), 17: Laukoter et al. (2017), 18: Na et al. (2008), 19: Saxena et al. (2008), 20: Steinbach et al. (2016), 21: Tröscher et al. (2019). 22: Machado‐Santos et al. (2018), 23: van Nierop et al. (2017), 24: Zrzavy et al. (2017). 25: Felts et al. (2005), 26: Marik, Felts, Bauer, Lassmann, and Smith (2007), 27: Nau and Brück (2002).

Such inflammatory diseases of the CNS have to be distinguished from conditions of “neuroinflammation,” which for instance occur in neurodegenerative or metabolic diseases (Becher et al., 2017; Dendrou, McVean, & Fugger, 2016; Hammond, Marsh, & Stevens, 2019). In these conditions brain injury is associated by immune reactions, which are associated with tissue injury. They mainly consist of reactions related to innate immune activation, involving resident monocytes, microglia, and astrocytes. Since also in these conditions immune cytokines and chemokines are locally liberated, recruitment of inflammatory cells from the blood, such as lymphocytes, granulocytes, and macrophages may occur. However, convincing evidence that in these conditions cells of the adaptive immunity are activated through local antigen recognition, with subsequent proliferation and clonal expansion is largely absent. Thus, on a quantitative neuropathological basis the lymphocytic inflammatory reaction in such conditions is marginal in comparison to that seen in classical inflammatory diseases of the brain and spinal cord (Machado‐Santos et al., 2018). Conditions of neuroinflammation and the respective consequences will be discussed in detail in other chapters of this special issue. For this reason, this review concentrates on the inflammatory CNS diseases proper.

## 2 THE SPECTRUM OF INFLAMMATORY DISEASES OF THE BRAIN AND SPINAL CORD REFLECTS THE BASIC IMMUNOLOGICAL MECHANISMS OF THEIR INDUCTION

The experimental model most commonly used to study CNS inflammation and the role of glia in this process is acute and chronic experimental autoimmune encephalomyelitis (EAE) in mice (Lassmann & Bradl, 2017) (Table 1). It is a disease, induced by active sensitization with brain antigens, such as for instance a peptide of myelin oligodendrocyte glycoprotein, or by passive transfer of CNS antigen reactive MHC Class II restricted CD4^+^ T‐lymphocytes (Ben‐Nun et al., 1981; Lassmann & Bradl, 2017). The advantages of this model are its convenience, its high reproducibility, and the technical ease to perform complex immunological studies in mice. The major disadvantages, however, are that it mimics only a very small part of the spectrum of human inflammatory diseases. Furthermore in most instances it is restricted to a single animal species (and strain) with a single immunization protocol. Already within EAE models, induced in different animal species with different immunization protocols, a much broader spectrum of brain inflammation is reproduced, which may provide experimental models, which are superior to address specific questions related to human disease (Lassmann & Bradl, 2017). In addition, in human pathology inflammatory diseases with T‐cell inflammation dominated by CD4^+^ T‐cells, the cells which trigger EAE, are rare. They include some EAE like diseases with features of acute disseminated leukoencephalomyelitis and Clippers syndrome (Blaabjerg et al., 2016; Höftberger & Lassmann, 2017a, 2017b).

Another type of brain and spinal cord inflammation involves the cooperation of T‐cells (most likely CD4^+^ cells) and specific pathogenic autoantibodies (Pohl et al., 2013). The classical examples of human diseases are neuromyelitis optica spectrum disorders with antibodies against the astrocytic water channel aquaporin 4 (Lennon, Kryzer, Pittock, Verkman, & Hinson, 2005) and the inflammatory demyelinating disease associated with specific demyelinating autoantibodies against myelin oligodendrocyte glycoprotein (Hennes, Baumann, Lechner, & Rostasy, 2018). For both diseases, excellent experimental models are available, including MOG induced experimental autoimmune encephalomyelitis in rats, guinea pigs, and marmoset monkeys (Lassmann & Bradl, 2017; Linington et al., 1988) and passive transfer models using aquaporin 4 specific T‐cells together with human or animal derived pathogenic autoantibodies against aquaporin 4 (Bradl et al., 2009; Hillebrand et al., 2019). The reaction of glia in these models not only depends upon the mechanisms of inflammation but also on the extent and type of antibody mediated tissue injury.

The vast majority of human acute and chronic inflammatory brain diseases, however, reflect an inflammatory reaction, massively dominated by MHC Class I restricted CD8^+^ T‐lymphocytes (Machado‐Santos et al., 2018). In some of these diseases, such as paraneoplastic encephalitis (Dauvilliers et al., 2013), Rasmussen's encephalitis (Bien et al., 2002), and viral encephalitis (Cox, Kahan, & Zajac, 2013; Laukoter et al., 2017), evidence for tissue injury and elimination of infected cells, which is directly mediated by cytotoxic CD8^+^ lymphocytes, has been provided. Only few groups so far have developed experimental models, which reflect brain inflammation by CD8^+^ T‐cells. Since it is very difficult to induce a CD8^+^ T‐cell mediated autoimmune response directly by active sensitization, such models are either induced by coupling a brain antigen with a virus vaccine (Ji et al., 2010) or by using a transgenic approach. In the latter, a foreign protein is expressed in transgenic animals in different cells of the central nervous system, such as astrocytes (Cabarrocas et al., 2003), oligodendrocytes (Na et al., 2008; Saxena et al., 2008), or neurons (Bernard‐Valnet et al., 2016). Disease is then transferred by the systemic injection of specific CD8^+^ T‐cell lines into the transgenic recipient animals. These models reproduce a variety of different human disease conditions, such as paraneoplastic encephalopathies, certain inflammatory demyelinating diseases, diabetes insipidus, narcolepsy and others. They have, however, so far not systematically analyzed regarding inflammatory mechanisms in the brain and the role and involvement of glia in this process.

A different situation is seen in multiple sclerosis, the most frequent inflammatory disease of the nervous system in the Western world. In this disease, the inflammatory reaction is dominated by CD8^+^ T‐cells (Babbe et al., 2000; Booss, Esiri, Tourtellotte, & Mason, 1983; Hayashi, Morimoto, Burks, Kerr, & Hauser, 1988) together with B‐cells (Blauth, Owens, & Bennett, 2015; Machado‐Santos et al., 2018) and recent clinical trials show effectivity of immunosuppressive agents that target T‐cells and B‐cells or even B‐cells alone, while specific therapies directed against CD4^+^ T‐cells have so far been ineffective (Lassmann, 2019). In contrast to the acute diseases mediated by cytotoxic Class I restricted T‐cells, the CD8^+^ T‐cells in MS show a dominant phenotype of inactive tissue resident effector memory cells with focal and temporally restricted activation (Machado‐Santos et al., 2018; van Nierop et al., 2017). Such T‐cell populations are mainly found experimentally in models of virus encephalitis, where such cells persist in the CNS as immune guardians after virus clearance and can be reactivated by re‐exposure to their cognate antigen (Schenkel & Masopust, 2014; Steinbach et al., 2016). Regarding B‐cells, CD19 and CD20 positive B‐cells dominate in active MS lesions and transform into immunoglobulin producing plasmablasts and plasma cells with lesion maturation (Frischer et al., 2009; Machado‐Santos et al., 2018). So far experimental models, defining the role of B‐cells in the induction and propagation of inflammation in the brain and spinal cord are missing.

Finally, acute bacterial meningitis and encephalitis represent a completely different aspect of the spectrum of inflammatory brain diseases. Here, the trigger of inflammation is not adaptive immunity, but the liberation of proinflammatory components from lysed bacteria and the activation of innate immune reactions in meningeal macrophages (Barichello et al., 2016; Nau & Brück, 2002). This results in recruitment of granulocytes and monocytes/macrophages and some lymphocytes into the meningeal compartment. Bacterial meningitis is associated with tissue injury, in particular in the outer cortical layers, which also results in activation of the local microglia and astrocyte population (Nau & Brück, 2002).

Thus, there is a very broad spectrum of human inflammatory diseases of the central nervous system, which occur on the basis of fundamentally different mechanisms of inflammation and immune mediated tissue injury (Table 1).

## 3 MICROGLIA IN THE NORMAL RODENT AND HUMAN BRAIN

Major progress has been achieved during the last years regarding the origin, phenotype, and function of microglia in the normal rodent brain. Microglia are tissue resident and self‐renewing cells in the central nervous system (Ajami, Bennett, Krieger, Tetzlaff, & Rossi, 2007), which enter the brain and spinal cord as yolk sac derived myeloid progenitors during fetal development and assume their mature phenotype in contact with specific environmental cues in the CNS (Alliot, Godin, & Pessac, 1999; Ginhoux et al., 2010; Kierndorf et al., 2013). Transforming growth factor beta (TGF‐ß) is one of the key cytokines mediating this transformation (Butovsky et al., 2014) and colony stimulating factor 1 (CSF‐1) is instrumental for the differentiation and survival of microglia in vivo (Greter et al., 2012; Wang et al., 2012). Thus, conditional genetic deletion of CSF‐1 or its pharmacological blockade results in efficient deletion of the cells from the tissue (Elmore et al., 2014). Within the normal CNS microglia assumes a resting or homeostatic phenotype, which is characterized by a cell type specific pattern of gene and protein expression (Butovsky et al., 2014; Gautier et al., 2012; Hickman et al., 2013). In conventional human neuropathology, this profile can be detected by the expression of three markers. Iba‐1 is a very reliable marker of all microglia and other myeloid cells in the CNS, but it is also expressed on bone marrow derived myeloid cells, recruited in pathological conditions. TMEM119 is a marker, selectively expressed in microglia, being absent from recruited myeloid cells (Satoh et al., 2016), and the purinergic receptor P2RY12 is a sensitive marker for the homeostatic phenotype of microglia (Butovsky et al., 2014). Its expression is downregulated under activation conditions for instance through the recognition of apoptotic cells through a TREM2/APOE dependent signaling cascade (Krasemann et al., 2017). The caveat for using TMEM119 for the identification of microglia derived cells is that it is also part of the homeostatic signature of microglia and its expression is down‐regulated under certain activation conditions (Krasemann et al., 2017).

Although the core signature of homeostatic microglia is basically preserved in the entire CNS, there are differences in the expression patterns of homeostatic genes during different stages of fetal development and in different CNS regions in the adult brain (Grabert et al., 2016; Michell‐Robinson et al., 2015; O'Loughlin, Madore, Lassmann, & Butovsky, 2018). This allows the differentiation of heterogeneous microglia subpopulations by single cell analysis, although these different cell phenotypes rather represent a transcriptional continuum instead of distinct cellular subtypes (Masuda et al., 2019). Overall, the expression of homeostatic microglia markers is most pronounced in the cerebral cortex and the hippocampus, more in gray compared to white matter and much more variable in the spinal cord. More recently it has been shown that in addition to microglia also perivascular and meningeal monocyte like cells are derived from yolk sac progenitors (Goldmann et al., 2016). However, even minor pathological or metabolic insults in the absence of overt tissue damage, such as for instance CNS irradiation, may lead to a major replacement of perivascular monocytes and a variable amount of cells with a microglia phenotype by recruited bone marrow derived myeloid cells (Hickey & Kimura, 1988; Lassmann, Schmied, Vass, & Hickey, 1993; Mildner et al., 2007). The dominant replacement of perivascular monocytes by recruited myeloid cells has also been confirmed in humans with sex mismatched bone marrow transplantation (Unger et al., 1993).

The phenotype of microglia in the normal human brain is much more complex. Although the core signature of homeostatic microglia is also preserved in the human brain, in most cases microglia show a partially activated phenotype even in the absence of any other neuropathological alterations (Wimmer, Zrzavy, & Lassmann, 2018). In particular, subsets of the cells already express markers for phagocytic activation, for oxygen radical productions, such as for instance NADPH oxidase (CYBB) and for antigen presentation (MHC Class I and Class II antigens and costimulatory molecules) and these activation markers are even expressed in microglia, which still show otherwise a homeostatic marker profile with reactivity for TMEM119 and P2RY12 (Wimmer et al., 2018; Zrzavy et al., 2017). This is also reflected in gene expression, revealing additional and more complex clusters of microglia phenotypes (Masuda et al., 2019) and by different age related gene expression patterns between mice and humans (Galatro et al., 2017). Another difference between rodent and human microglia is the abundance of dystrophic or senescent microglia, containing iron or ferritin in humans (Lopes, Sparks, & Streit, 2008; Streit, Sammons, Kuhns, & Sparks, 2004), which appears to be related to age dependent iron accumulation in the normal human brain (Hallgren & Sourander, 1958; Hametner et al., 2013).

The reason for this diverse phenotype of microglia in the normal human brain is so far only poorly understood. One factor may be that humans, in contrast to laboratory rodents, are more extensively exposed to systemic infections. Systemic sepsis in humans in the absence of brain infection or neuropathological lesions is associated with a proinflammatory activation of microglia mainly in the cerebral white, but less in the gray matter (Shimada & Hasegawa‐Ishii, 2017; Zrzavy et al., 2019). Microglia showed an activated morphological phenotype with increased cytoplasmic volume and reduced cell processes. They still expressed the microglia marker TMEM119 and the homeostatic marker P2RY12, but revealed a transitional phenotype with increased expression of all proinflammatory markers. Whether this is due to innate immune memory (Wendeln et al., 2018) has to be determined in the future.

Another possible explanation for microglia activation in the “normal” human brain is the more pronounced age related neurodegeneration in comparison to that in rodents. Thus, a correlation between loss of homeostatic phenotype of microglia with the presence of neurofilament changes in axons and neurons was not only seen in the cortex of Alzheimer's disease patients, but also in the cortex of age matched controls (Krasemann et al., 2017). Other factors, which may affect the state of microglia activation in the normal brain are exposure to environmental toxins or diet and the composition of the gut microbiome (Erny et al., 2015). All these factors are kept constant as much as possible in the environment of laboratory animals, but are apparently highly variable in humans. Thus, in contrast to rodent models microglia activation in human inflammatory CNS diseases develop on the background of a preactivated or dystrophic microglia phenotype, and this may have major implications for the functional state of the cells during lesion development or repair (Perry & Holmes, 2014).

## 4 MICROGLIA AND MACROPHAGES IN INFLAMMATORY DISEASES OF RODENTS

Numerous studies have shown microglia and macrophage activation in the inflammatory brain lesions of experimental autoimmune encephalomyelitis in rodents. Microglia activation has been described to precede clinical disease and T‐cell infiltration in this model (Grygorowicz & Struzynska, 2019; Ponomarev, Shriver, Maresz, & Dittel, 2005), although it has not been taken into account that T‐cells infiltrate the meninges very early in the disease and that this precedes the appearance of parenchymal inflammatory lesions (Flügel et al., 2001).

Already in the earliest stages of the inflammatory process a loss of the homeostatic phenotype is seen (Krasemann et al., 2017), which is associated with a downregulation of the relevant gene expression, but also with shedding of some of the homeostatic markers from the surface of activated cells. This goes along with a transition of resting microglia into an amoeboid phenotype and the appearance of cells with macrophage morphology. With time after onset of the inflammatory reaction there is an increase in the expression of activation markers, which include proteins involved in phagocytosis, antigen presentation, and costimulation (Schuh et al., 2014) and this is also associated with increased expression of proinflammatory cytokines (Wang, Asensio, & Campbell, 2002). During the recovery process the proinflammatory activity declines and this is associated with a transition of the cells into an anti‐inflammatory or repair promoting phenotype (Gao & Tsirka, 2011; Kwidzinski et al., 2005; Ransohoff, 2016). Although these basic patterns of microglia and macrophage activation are already well established, the exact contribution of microglia versus recruited myeloid cells and the possible presence of different myeloid cell subpopulations in the lesions remained controversial. Early studies, using models of immune ablation and bone marrow transplantation showed a very prominent contribution of recruited cells to the total myeloid cell population at the sites of inflammation, while microglia activation was mainly present in the adjacent normal appearing white or gray matter tissue (Hickey & Kimura, 1988; Lassmann et al., 1993).

The dynamic changes of microglia activation and the contribution of recruited myeloid cells in the course of lesion development and resolution has recently been studied by single‐cell‐mass cytometry (CYTOF), which allows to determine the dynamic changes of different cell populations by a combined marker profile (Ajami et al., 2018). The study confirms earlier work discussed above by showing that already in the preclinical phase of the disease microglia become activated and circulating monocytes are recruited into the inflammatory lesions. In addition, it shows that there are several different subpopulations of microglia and of recruited monocytes present, which can be distinguished by their patterns of marker expression. However, they do not represent specific microglia or macrophage subtypes, but rather a continuum in the activation process. The prominent expression of MHC Class II molecules and the production of various proinflammatory cytokines in the disease associated microglia and monocyte populations suggested a prominent role in the inflammatory disease process. However, since this study was performed by isolating the cells from the entire brain or spinal cord, no information is provided regarding the relative contribution of microglia and recruited monocytes in inflammatory foci versus the normal brain and spinal cord tissue. Furthermore, an exact designation of different microglia or monocyte subtypes to specific types and stages of pathology or the relation of them to specific types of tissue injury was not possible.

The important role of microglia and/or recruited monocytes in the induction and propagation of the inflammatory response is supported by the observation, that macrophage/microglia inhibition attenuates EAE (Cuzner & Opdenakker, 1999; Nissen, Thompson, West, & Tsirka, 2018) and long term monocyte depletion after disease onset prevents disease progression and accumulation of axonal damage (Moreno et al., 2016). These effects may be due to blockade or depletion of microglia, perivascular monocytes in the brain or recruited myeloid cells. Using radiation bone marrow chimeric animals and disease induction with MHC mismatched donor T‐cells, it was shown that antigen presentation on CNS resident microglia and monocytes is not necessary for disease induction (Hickey & Kimura, 1988). This has recently been confirmed using transgenic mouse lines with specific deletion of MHC Class II antigens in microglia (Wolf et al., 2018). Brain inflammation is induced by the antigen‐specific interaction of T‐cells with meningeal and perivascular antigen presenting cells (Bartolomäus et al., 2009; Flügel et al., 2001; Mues et al., 2013). In EAE models, these cells have not to be derived from the resident pool of perivascular monocytes, but it is sufficient when these cells are recruited perivascular and meningeal myeloid cells. Although these cells express some markers, typical for dendritic cells (Greter et al., 2005; Mundt et al., 2019) they may not be genuine dendritic cells, since dendritic cell antigens may be induced on macrophage populations due to environmental cues (Hochmeister et al., 2008).

Taken together, antigen recognition by T‐cells on resident myeloid cells of the brain is not required for the induction of inflammation by CD4^+^ T‐lymphocytes; however, microglia may be involved in the induction or propagation of immune mediated tissue injury. To address this question, EAE was induced in experimental models, where microglia was depleted or activated in a proinflammatory phenotype at the time, when inflammatory lesions were initiated in the brain or spinal cord by auto‐reactive CD4^+^ T‐cells. In one study (Heppner et al., 2005), the immune activation was specifically prevented in microglia at the time of EAE induction. In this model, a moderate reduction of EAE severity was noted, associated with decreased brain inflammation. One caveat in this experiment is that ganciclovir treatment alone may ameliorate EAE (Ding et al., 2014). In another model, microglia were rapidly depleted by diphtheria toxin in a transgenic model of microglia specific expression of the diphtheria toxin receptor (Rubino et al., 2018). This resulted in nearly complete microglia loss within the first days after toxin treatment and was followed by an overshooting microglia repopulation associated with neurodegeneration by proinflammatory activation of microglia. EAE induction was timed in a way that the onset of inflammation occurred either at the time of microglia depletion or of overshooting microglia activation. Neither of these conditions had an effect on EAE related inflammation nor on the neurodegenerative process due to the pre‐existing microglia activation (Rubino et al., 2018). In a further study, EAE was induced by active immunization or passive T‐cell transfer in an experimental model, which was characterized by microglia activation, accelerated iron accumulation in the brain, and progressive neurodegeneration (Wimmer et al., 2019), a pathological scenario which closely reflects the tissue changes seen in the progressive stage of multiple sclerosis (Mahad, Trapp, & Lassmann, 2015). Also in this model, the pathology of EAE was not modified by the pre‐existing microglia activation, nor was the progressive neurodegenerative phenotype altered by the inflammatory process of EAE. However, inflammatory infiltrates were preferentially located at sites of microglia activation and pre‐existing brain damage. Finally, in another study EAE was induced in animals with cuprizone related demyelinating lesions in a timing, which resulted in the appearance of inflammatory lesions at sites of microglia activation, active demyelination, and neurodegeneration in the corpus callosum. Similarly to the previous study, inflammatory lesions were preferentially precipitated to the side of microglia activation and brain damage, but the EAE typical pattern of brain inflammation or the extent of tissue damage was neither altered by the presence of inflammation at the site of microglia activation nor by the presence of microglia preactivation at the site of EAE‐induced brain inflammation (Ruther et al., 2017).

Thus so far there is no compelling evidence that tissue resident microglia or monocytes are essential for inflammation or immune mediated tissue injury in EAE, induced by MHC Class II restricted CD4^+^ T‐cells, but that all disease‐relevant pathologies can be triggered by the interaction between CD4^+^ T‐cells and recruited myeloid cells. This does not exclude that more subtle disease modifying effects may be seen in future experiments. However, these findings also raise the question, whether the commonly used model of CD4^+^ T‐cell dependent EAE is suitable to study the role of microglia and other tissue resident myeloid cells in relation to human inflammatory diseases of the CNS. As mentioned above, in most human inflammatory brain diseases inflammation is dominated by CD8^+^ T‐cells or, less frequently by B‐cells (Machado‐Santos et al., 2018). For such conditions, experimental models are rare at present and only sparse data on microglia and macrophages are available. The pathology of brain or spinal cord mediated inflammation in experimental models driven by CD8^+^ T‐cells suggest that in these conditions microglia activation is prominent, while recruitment of macrophages into the lesions is sparse (Cabarrocas et al., 2003). So far, however, no detailed phenotypic analysis of the microglia and monocyte populations in these models is available. Even less is known about the relation between B‐cells in inflammatory brain lesions and the patterns of astrocyte and microglia activation patterns.

## 5 MICROGLIA AND MACROPHAGES IN INFLAMMATORY DISEASES IN HUMANS

**Figure 1:**
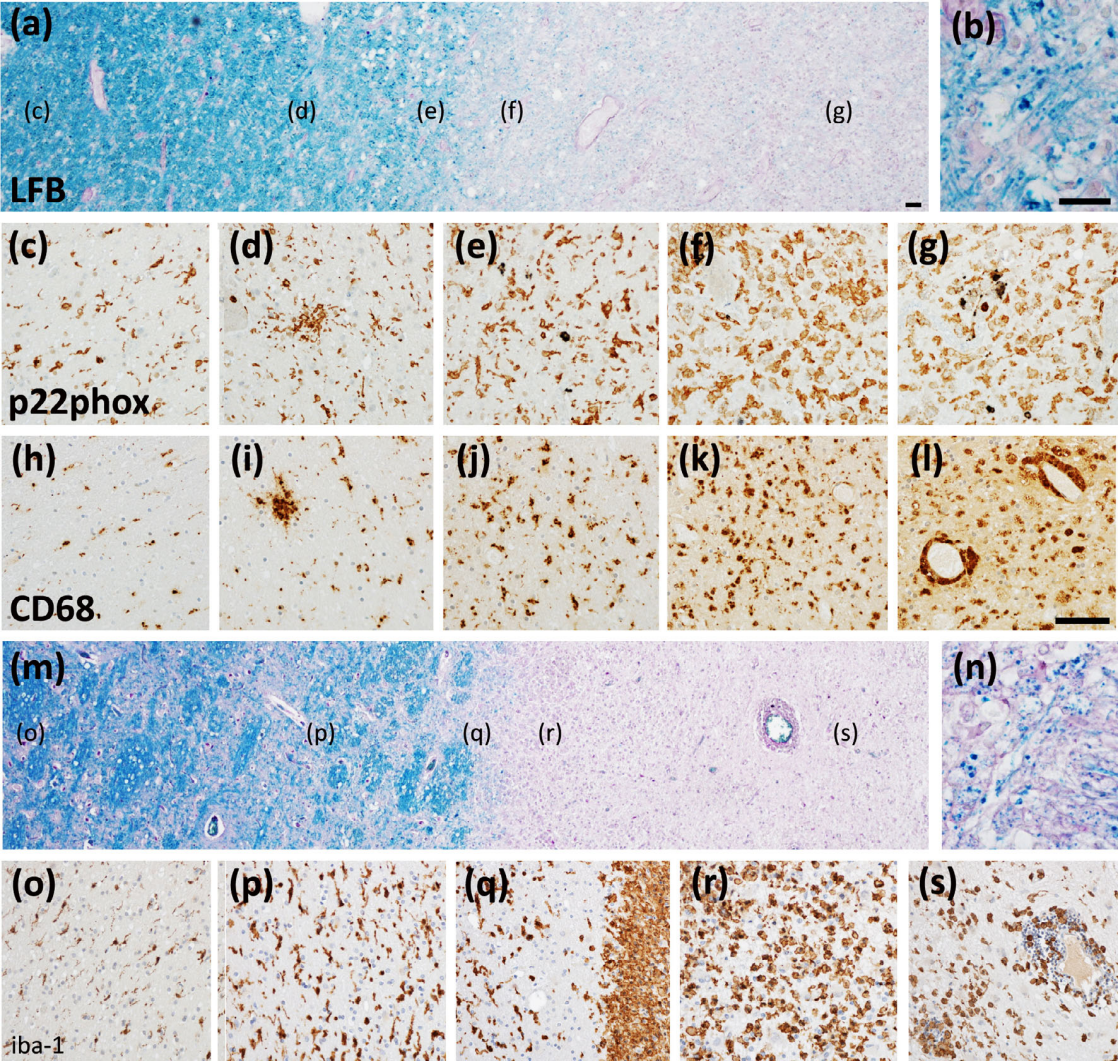
The pattern of microglia activation in inflammatory brain lesions depends upon the mechanisms of tissue injury and the type and stage of the lesion. (a–l) Active lesion in a patient with acute inflammatory demyelinating disease fulfilling the pathological diagnostic criteria of multiple sclerosis (confluent inflammatory demyelinating lesion with axonal preservation) with profound oxidative injury and mitochondrial damage (Pattern III, Lucchinetti et al., 2000): (a) the demyelinated lesion shows a transition zone between the normal appearing white matter and the actual demyelinated areas with an increasing density of activated microglia and macrophages, expressing besides the pan microglia marker Iba‐1 very prominently also NADPH‐oxidase (p22phox); (b) higher magnification of the lesion edge shows the presence of granular Luxol fast blue reactive myelin degradation products in macrophages; (c, h) the normal appearing white matter reveals a normal density of microglia, but they already express NADPH oxidase (p22phox; c) and the lysosomal marker CD68 (h); (d, i) in the peri‐plaque white matter there is a general activation of microglia with the formation of small microglia nodules; (e, j) in the zone of initial demyelination ongoing myelin damage is associated with a profound microglia activation and expression of markers for oxidative injury (p22phox) and phagocytosis (CD68); (f, k) in early active lesions myelin is destroyed and the remnants are taken up in cells with macrophage morphology; (g, l) in late active lesions a reduced expression of NADPH oxidase is seen in macrophages, which in part accumulate in the perivascular space. (m–s) Active lesion in a patient with acute inflammatory demyelinating disease fulfilling the pathological diagnostic criteria of multiple sclerosis (as in the images a–l) but with antibody and complement mediated myelin destruction (Pattern II, Lucchinetti et al., 2000). (m) There is a sharp border between the demyelinated plaque and the adjacent white matter; (n) higher magnification shows the presence of myelin degradation products in macrophages at the lesion edge; (o) normal appearance of microglia in the normal appearing white matter; (p) increased numbers of activated microglia in the peri‐plaque white matter adjacent to the lesion; (q) the lesion edge shows a small zone of reduced microglia density adjacent to a massive infiltration of the tissue by cells with macrophage morphology, which contain early myelin degradation products; (r) in the early active zone myelin is destroyed and the macrophages contain early myelin degradation products; (s) in the late active/inactive center there is a much lower density of macrophages and these cells in part accumulate in the perivascular space of inflamed vessels. Magnification bar: 100 μm

Multiple sclerosis is the most common chronic human inflammatory disease of the central nervous system in the Western World and therefore much of our knowledge on microglia and macrophages comes from studies of this disease. Multiple sclerosis is a chronic inflammatory demyelinating disease of the CNS (Lassmann et al., 2007). In general its starts in young adults with a relapsing remitting disease course (RRMS), which is followed after 10–15 years of disease duration by a phase of slow and uninterrupted disease progression (SPMS). Ten to 15% of the patients miss the relapsing disease stage and show disease progression from the onset (a disease phenotype called primary progressive MS; PPMS). A very small proportion of patients present with an acute and fulminant disease, which leads to the patient's death within the first year after disease onset. The pathology of MS is defined by the presence of inflammatory demyelinating lesions of different activity stages, which are surrounded by areas of normal appearing white matter (NAWM, Frischer et al., 2015, Kuhlmann et al., 2017). Active lesions show areas of acute myelin destruction, containing abundant macrophages with early myelin degradation products. Inactive lesions can still contain macrophages with lipid inclusions as remnants of the degraded myelin, but the majority is devoid of macrophages. Remyelination can be seen already in some active lesions (Prineas, Barnard, Kwon, Sharer, & Cho, 1993), and ongoing remyelination is most prominent at sites, which are still infiltrated by macrophages. MS lesions occur both in the white and gray matter. Active white matter lesions are in general characterized by profound inflammation, while in the gray matter inflammation is less pronounced and frequently restricted to the adjacent meninges. In the progressive stage of the disease classical active lesions, as described above, become rare (Frischer et al., 2015). The most frequent lesions are inactive plaques. Ongoing activity is seen in a subset of lesions, called chronic active or slowly expanding lesions (Kuhlmann et al., 2017; Prineas et al., 2001). They consist of an inactive lesion core, which is surrounded by a rim of activated microglia, associated with a low degree of ongoing myelin and axonal destruction. When discussing the glia reaction in MS lesions, the complex architecture of the lesions has to be taken into account (Lassmann, 2011; Figure 1).

Activated microglia and macrophages are abundant in active MS lesions (Esiri & Reading, 1987) and they are associated with ongoing demyelination (Brück et al., 1995) and progressive axonal injury (Ferguson, Matyszak, Esiri, & Perry, 1997). Phenotypic changes of microglia and macrophages in the different portions of active lesions suggest a temporal and spatial evolution of microglia activation in relation to tissue injury (Bogie, Stinissen, & Hendriks, 2014; Peferoen et al., 2015;Vogel et al., 2013 ; Zrzavy et al., 2017). Microglia phenotype in the normal appearing white matter of MS patients in early disease stages is closely similar to that in the normal age matched human brain. It consists of microglia, which to a large extent expresses homeostatic markers, but which simultaneously express antigens related to proinflammatory activation (Zrzavy et al., 2017). This proinflammatory activation is more pronounced in the normal appearing white matter of patients with progressive MS than with RRMS. The increased microglia activation in the NAWM may be due in part to secondary Wallerian degeneration, which occurs in the NAWM of such patients as a consequence of axonal transection in focal demyelinated lesions in the white matter or the cortex (Dziedic et al., 2010). In addition, the NAWM in MS patients in the progressive stage of the diseases frequently contains perivascular inflammatory infiltrates, suggesting diffuse immune mediated damage in the entire brain and spinal cord (Kutzelnigg et al., 2005).

The proinflammatory microglia activation reaches its peak at the edge of actively demyelinating lesion (Marik et al., 2007). In this area initial demyelination and neurodegeneration occurs, reflected by oligodendrocyte apoptosis and degeneration of oligodendrocyte processes (Lassmann, 2011). More than 90% of the myeloid cells in this location express the microglia marker TMEM119, but the expression of homeostatic markers (P2RY12) is lost. Furthermore, increased reactivity for markers related to phagocytosis, oxidative injury, antigen presentation, and costimulation are seen (Haider et al., 2011; Zrzavy et al., 2017). A similar proinflammatory activation is present in myeloid cells in active lesions, where myelin is already destroyed and ingested by activated macrophages (early active lesions; Brück et al., 1995). To what extent these macrophages come from the local microglia pool or from recruited bone marrow derived myeloid cells is not fully clear yet. The fact that in the initial stages of demyelination microglia and a large proportion of cells with macrophage phenotype are TMEM119 positive (Zrzavy et al., 2017) suggests that microglia and not the hematogenous macrophages play a critical role in the initiation of the demyelinating process in MS (Satoh et al., 2016). However, with lesions maturation TMEM119 expression is increasingly lost, which possibly is due to a dilution of original microglia derived cells by recruited monocytes or to the progressive loss of TMEM119 as a homeostatic microglia marker (Krasemann et al., 2017). With further maturation of the lesions, a change in macrophage polarization occurs, which may be related to the phagocytosis and digestion of myelin components (Boven et al., 2006). These phagocytes present with a phenotype, which is intermediate between proinflammatory (M1) and anti‐inflammatory (M2) activation (Vogel et al., 2013; Zrzavy et al., 2017). They still express markers associated with propagation of inflammation and active tissue injury, but also produce and express molecules, associated with anti‐inflammatory polarization and with the propagation of tissue repair.

The pattern of microglia activation in the slowly expanding lesions in patients with progressive MS is different from that in classical active lesions. Actively expanding lesions are demarcated from the NAWM by a rim of activated microglia, which dominantly expresses proinflammatory markers. Polarization of these cells into an intermediate or anti‐inflammatory phenotype is rare or absent. In the inactive center of the lesions, no macrophages are present and even the density of microglia within the tissue is much lower, compared to that in the normal appearing white matter (Zrzavy et al., 2017).

The reason for the loss of microglia in the inactive center of MS lesions is so far not clear. In part it may be due to the fact that microglia in active lesions transform into macrophages and are involved in the uptake and degradation of myelin. Macrophages with myelin degradation products are originally dispersed throughout the whole lesion, but with time accumulate in the perivascular spaces and may possibly be drained into the meninges, as suggested already by Marburg (1906). In addition, in the brain, but not in the spinal cord microglia take up iron and ferritin (Hametner et al., 2013; Mehta et al., 2013), and microglia dystrophy or senescence is mainly seen in iron and ferritin containing cells, which are more prominent in MS patients than in controls (Hametner et al., 2013; Lopes et al., 2008). Iron containing microglia at the lesion edge indicates lesion expansion in patients with progressive MS. Such iron rims around lesions can even be detected in the living patient by high field magnetic resonance imaging (Dal Bianco et al., 2017).

Experimental studies have provided evidence that macrophages, which are polarized toward an anti‐inflammatory phenotype, can promote remyelination and repair in demyelinated lesions (Miron et al., 2013; Miron & Franklin, 2014). In line with this observation, active remyelination in MS lesions is most prominently in active lesions with a high content of macrophages with intermediate polarization. In contrast, no or very sparse remyelination is seen in slowly expanding lesions in patients with progressive MS (Bramow et al., 2010), where only activated microglia with a proinflammatory phenotype are present (Zrzavy et al., 2017).

In a recent study, an attempt was made to characterize the microglia and macrophage response in MS lesions through single cell gene expression. Although this approach allows the differentiation of bone marrow derived cells from genuine microglia and of different subsets of microglia and monocytes, it so far only produced evidence for the existence of different cell populations in the lesions, but did not provide any insights into their potential role in inflammation or tissue injury (Masuda et al., 2019). All presently available data document very complex spatial and temporal patterns of the microglia and macrophage response in the dynamic evolution of MS lesions at different disease stages (Lassmann, 2011, Figure 1). Thus single cell fate mapping in such a condition only makes sense, when cells are specifically analyzed in relation to lesion type and stage.

In comparison to MS information about microglia in other human inflammatory diseases of the central nervous system is less complete (Terry et al., 2012). Overall, both, pro and anti‐inflammatory roles have been suggested. However, some additional more specific mechanisms have been described. Microglia cells apparently are very effective sensors for infection related cell damage and respond with inflammasome activation for instance during HIV infection (Katuri, Bryant, Heredia, & Makar, 2019; Mamik et al., 2017). A similar mechanism has recently been observed in Rasmussen's encephalitis, an inflammatory and immune mediated epilepsy, driven by MHC Class I restricted cytotoxic CD8^+^ T‐cells, which directly interact with neurons and astrocytes (Bauer, Gold, Adams, & Lassmann, 2015; Bien et al., 2002; Dauvilliers et al., 2013). The initial step in the development of lesions in this disease is the formation of microglia nodules with inflammasome activation, which precedes the CNS infiltration by T‐cells (Tröscher et al., 2019). Interestingly, such microglia nodules are also abundant in the normal appearing white matter adjacent to active lesions in MS (Michailidou et al., 2017; Peferoen et al., 2015; Singh et al., 2013; van der Valk & Amor, 2009, Figure 1). The early inflammasome activation of microglia appears to be an important general mechanism of infection control in the CNS. There is agreement between studies, that microglia depletion by pharmacological inhibition of the CSF‐R1 exacerbates disease in diverse models of virus infection of the brain (Sanchez et al., 2019; Seitz, Clarke, & Tyler, 2018; Waltl et al., 2018; Wheeler & Quintana, 2019). This may be due to a role of microglia in the local restimulation of CD8^+^ T‐cells (Funk & Klein, 2019). In addition, microglia are attracted by nucleotide release from damaged neurons, recognized by their purinergic receptors, and reduce the spread of infection by isolating the infected cells (Fekete et al., 2018).

In certain virus infections of the CNS, such as for instance in HIV infections, microglia are by themselves infected and propagate the infection in the brain and spinal cord (Burdo, Lackner, & Williams, 2013). As an example, “humanized” bone marrow chimeric mice are susceptible to brain infection by HIV, when the transplanted cells develop into cells with microglia properties within the CNS (Mathews et al., 2019). In addition, as shown by single cell gene expression analysis, chronic microglia infection in the AIDS brain may result in their functional impairment and senescence, which may impair their homeostatic function in chronic brain inflammation (Chen, Partridge, Sell, Torres, & Martin‐Garcia, 2017; Ginsberg et al., 2018).

## 6 ASTROCYTES IN EXPERIMENTAL AND HUMAN INFLAMMATORY BRAIN DISEASE

Astrocytes play a major role in the induction and modification of brain inflammation, which is summarized and discussed in a number of excellent recent review articles (Brambilla, 2019; Ludwin, Rao, Moore, & Antel, 2016; Ponath, Park, & Pitt, 2018; Soung & Klein, 2018; Wheeler & Quintana, 2019). For this reason, only few aspects will be addressed here, which go beyond the issues covered in detail in these articles. Like microglia, and in close cooperation with them, astrocytes apparently are activated in initial stages of inflammatory diseases (Liddelow et al., 2017) and this process is in part controlled by products of the gut microbiome (Rothhammer et al., 2018). As a prominent source of cytokines, chemokines and other inflammatory mediators they are instrumental both in the propagation as well as in the downregulation of inflammatory responses in the brain. Due to their strategic location at the blood brain interface they are important for blood brain barrier function and in particular in controlling the spread of inflammatory cells from the perivascular space into the parenchyma (Brambilla, 2019). They further promote T‐cell proliferation and expansion by antigen presentation via the expression of MHC Class I and Class II molecules or costimulatory molecules (Höftberger et al., 2004). NFkB activation in astrocytes, but less in microglia is associated with demyelination and axonal injury in the cuprizone model and can be prevented by treatment with the anti‐inflammatory drug laquinimod (Brück et al., 2012). In later stages of the inflammatory process astrocytes become a prominent source of anti‐inflammatory or immune regulatory molecules, such as interleukin 10 or TGF‐ß, as even shown in the human brain (Machado‐Santos et al., 2018). In line with these observations, polarization into proinflammatory A1 and anti‐inflammatory A2 astrocytes, which is similar to M1 and M2 polarization of microglia, has been described in vitro and in vivo (Liu, Yang, Ju, Wang, & Thang, 2018), although with different profiles between mouse and human astrocytes (Tarassishin, Suh, & Lee, 2014).

An aspect, which received less attention, is the reaction of astrocytes to the inflammatory stimulus. In both, rodents and humans, a basic reaction pattern has been described, consisting of astrocyte activation in the early stages, leading to large activated astrocytes with massively increased cell volume, reduction of cell processes, and massive cytoplasmic accumulation of GFAP. This is followed at later stages by the formation of a glial scar, consisting of small astrocytes with cell processes, which are densely packed GFAP positive intermediate filaments. The glial scar forms a barrier, separating the lesion form the adjacent normal appearing white matter. It was argued for long that the glial scar formation is one of the reasons, why regeneration is blocked in lesions in the central nervous system and that this is further augmented by their expression of molecules, which inhibit progenitor cell migration or regenerating axons into the defect. This, however, has recently been questioned in experiments that show that the glial scar is an essential scaffold for axonal regeneration after spinal cord injury (Anderson et al., 2016).

In inflammatory brain lesions, components of the innate immune response may induce astrocyte dysfunction. Focal injection of lipopolysaccharide into the CNS tissue leads to an early inflammatory response, which is followed by a delayed phase of tissue injury with demyelination and axonal injury (Felts et al., 2005). Interestingly, the earliest change related to tissue injury is a functional disturbance of astrocytes. The cells become large and highly reactive for GFAP, but they lose their cell processes and their polarity. Thus the perivascular glia limitans becomes disturbed and the molecules, which are associated with astrocytic function at the glia limitans, such as aquaporins or α‐dystroglycan and connexins are lost (Sharma et al., 2010). This is then associated with a propagation of the inflammatory response and immune mediated demyelination and axonal damage. A very similar astrocyte injury has been observed in highly inflammatory lesions in EAE (Mayo et al., 2016) and in multiple sclerosis lesions, occurring at early disease stages (Sharma et al., 2010), and in an even more prominent form in the lesions of Balo's concentric sclerosis (Matsushita et al., 2011). Gene expression profiling of such lesions and their immunohistochemical analysis suggest that oxidative injury is the driving force for these astrocyte alterations (Fischer et al., 2012, 2013). Similar, but much more severe astrocytic changes are seen in the lesions in patients with neuromyelitis optica spectrum disorders (Misu et al., 2013). In these patients, astrocyte damage is induced by specific autoantibodies against the astrocytic water channel aquaporin‐4. This leads to a primary inflammatory astrocytopathy, followed by demyelination and a variable extent of axonal injury. The pathogenesis of this condition has been reproduced and characterized in detail in recent experimental studies, transferring pathogenic human aquaporin‐4 autoantibodies into rodents with a background of T‐cell mediated brain inflammation (Bradl et al., 2009; Bradl & Lassmann, 2014).

## 7 CONCLUSIONS AND OPEN QUESTIONS

Major progress has been made in the understanding of the role of glia cells, in particular astrocytes and microglia, in inflammatory conditions of the CNS. Much of this knowledge has been obtained through studies in the model of autoimmune encephalomyelitis in rodents. These studies revealed basic principles, including the transformation from homeostatic function of resident cells toward the early activation of microglia and astrocytes in the initial stages of the inflammatory process and their contribution both as pro‐ as well as anti‐inflammatory cells in the dynamic evolution of the inflammatory lesion. Although these basic principles are similar between laboratory rodents and humans, direct conclusions from experimental studies to specific human diseases should be done with caution for several reasons:

1. Although the core signatures of microglia and astrocytes homeostasis and activation are conserved between species, including rodents and humans, differences have been described in studies based on protein expression by immunohistochemistry as well as in gene expression studies (Wimmer et al., 2018). These differences are seen in the normal brain and spinal cord, in relation to brain aging, but also in inflammatory conditions. Such differences between humans and laboratory rodents may be due to genetic species differences, environmental differences or differences in the pathogenesis of the inflammatory diseases, investigated in humans versus in animal models.
2. Experimental models of inflammatory diseases in rodents develop on a background of truly resting or homeostatic microglia and astrocytes in a normal CNS tissue. It is an attractive hypothesis that microglia preactivation may facilitate chronic disease progression, as seen in progressive MS, but this was so far not seen in EAE models driven by CD4^+^ T‐cells.
3. Most studies on glia activation in brain inflammation have been performed for practical reasons in very restricted mouse models of EAE, induced in a selected susceptible mouse strain by immunization with a MOG peptide, which targets only a single immune mechanism. This is fundamentally different to human inflammatory brain diseases, which are diverse and induced by a range of different immunological mechanisms. Alternative models are currently available, which involve different animal strains and species and different immune mechanisms, which will have to be analyzed in detail in future studies, despite of being more complex and difficult to handle.
  Active demyelination and neurodegeneration in multiple sclerosis and in autoimmune encephalomyelitis is invariably associated with the presence of activated macrophages and/or microglia (Brück et al., 1995; Ferguson et al., 1997) and blockade of macrophage function has been suggested as a therapeutic strategy for inflammatory brain diseases. So far, however, selective blockade of macrophage and microglia activation has not been established in multiple sclerosis treatment with the exception of a single phase II clinical trial, which showed a moderate beneficial effect of minocycline (Metz et al., 2017). To develop such a therapeutic strategy for MS patients, efficacy and safety have to be demonstrated in a broad spectrum of experimental models. In addition, the role of macrophages and microglia in brain inflammation is not only detrimental, but also beneficial.

## CONFLICT OF INTEREST

Hans Lassmann received honoraria for lectures and consulting from Novartis, Biogen, Sanofi Aventis, Merck, Roche and MEDDAY. There is no direct conflict of interest related to the content of this manuscript.
